# Gene expression patterns and sequence polymorphisms associated with mosquito resistance to *Bacillus thuringiensis israelensis* toxins

**DOI:** 10.1186/1471-2164-15-926

**Published:** 2014-10-23

**Authors:** Laurence Després, Renaud Stalinski, Guillaume Tetreau, Margot Paris, Aurélie Bonin, Vincent Navratil, Stéphane Reynaud, Jean-Philippe David

**Affiliations:** Université Grenoble Alpes, Laboratoire d’Ecologie Alpine UMR5553, Grenoble, France; Centre National de la Recherche Scientifique (CNRS), Laboratoire d’Ecologie Alpine UMR5553, Grenoble, France; Pôle Rhône Alpes de Bioinformatique, Université Lyon 1, Lyon, France; Laboratoire d’Ecologie Alpine, 2233 rue de la piscine, Université J. Fourier, BP53, 38041 Grenoble Cedex 09, France

**Keywords:** RNA-seq, *Bacillus thuringiensis israelensis* toxins, Mosquito, Bio-insecticide resistance, Toxin receptors, Lipid rafts, Resistance costs, Evolutionary trade-offs

## Abstract

**Background:**

Despite the intensive use of *Bacillus thuringiensis israelensis* (Bti) toxins for mosquito control, little is known about the long term effect of exposure to this cocktail of toxins on target mosquito populations. In contrast to the many cases of resistance to *Bacillus thuringiensis* Cry toxins observed in other insects, there is no evidence so far for Bti resistance evolution in field mosquito populations. High fitness costs measured in a Bti selected mosquito laboratory strain suggest that evolving resistance to Bti is costly. The aim of the present study was to identify transcription level and polymorphism variations associated with resistance to Bti toxins in the dengue vector *Aedes aegypti*. We used RNA sequencing (RNA-seq) for comparing a laboratory-selected strain showing elevated resistance to Bti toxins and its parental non-selected susceptible strain. As the resistant strain displayed two marked larval development phenotypes (slow and normal), each phenotype was analyzed separately in order to evidence potential links between resistance mechanisms and mosquito life-history traits.

**Results:**

A total of 12,458 genes were detected of which 844 were differentially transcribed between the resistant and susceptible strains. Polymorphism analysis revealed a total of 68,541 SNPs of which 12,571 SNPs exhibited more than 40% frequency difference between the resistant and susceptible strains, affecting 2,953 genes. Bti resistance is associated with changes in the transcription level of enzymes involved in detoxification and chitin metabolism. Among previously described Bti-toxin receptors, four alkaline phosphatases (ALPs) were differentially transcribed between resistant and susceptible larvae, and non-synonymous changes affected the protein sequence of one cadherin, six aminopeptidases (APNs) and four α-amylases. Other putative Cry receptors located in lipid rafts, such as flotillin and glycoside hydrolases, were under-transcribed and/or contained non-synonymous substitutions. Finally, immunity-related genes showed contrasted transcription and polymorphisms patterns between the two developmental resistant phenotypes, suggesting the existence of trade-offs between Bti-resistance, life-history traits and immunity.

**Conclusions:**

The present study is the first to analyze the whole transcriptome of Bti-resistant mosquitoes by RNA-seq, shedding light on the importance of studying both transcription levels and sequence polymorphism variations to get a comprehensive view of insecticide resistance.

**Electronic supplementary material:**

The online version of this article (doi:10.1186/1471-2164-15-926) contains supplementary material, which is available to authorized users.

## Background

Biological control strategies involving *Bacillus thuringiensis israelensis* (Bti) are increasingly used as alternatives to chemicals in order to limit the spreading of mosquito-borne pathogens transmission and nuisance. During sporulation, Bti produces a composite crystalline inclusion composed of four main toxins: Cry4Aa, Cry4Ba, Cry11Aa and Cyt1Aa. Upon larval ingestion, the crystalline inclusions dissolve in the alkaline pH of the insect’s gut, releasing Cry and Cyt protoxins that will be processed to active toxins by insect gut proteases [1]. Cyt and Cry toxins are known to act in synergy to kill mosquito larvae, but the precise mechanisms of their interaction are not fully understood [2, 3]. Other *Bacillus thuringiensis* subspecies produce Cry toxins that are specific to their target organism, and most research has been done on Cry toxins mode of action in various insects (mostly in lepidopterans and coleopterans, but also in dipterans) and nematodes [4, 5]. The selectivity of Cry toxins is mainly due to the interaction with specific receptors present at the surface of midgut epithelium cells of the larva. The activated Cry toxins bind to specific protein receptors, insert into the membrane and form pores, resulting in midgut disruption, bacterial infection and death of the insect. In the recent years, several models have been proposed to describe the precise mode of action of Bt Cry toxins (reviewed in [6, 7]). The binding of Cry toxins can involve interaction with multiple specific receptors, including cadherin-like proteins but also aminopeptidases N (APNs) and alkaline phosphatases (ALPs) which are mostly located in lipid rafts (reviewed in [8]). In mosquitoes, besides cadherins, APNs and ALPs, α-amylases were also shown to bind to Cry4Ba and Cry11Aa toxins [9]. As compared to what is known about the mode of action of Bt Cry toxins in lepidopterans and coleopterans, the understanding of Bti mode of action in mosquito lies far behind, mainly due to the lack of Bti-resistant mosquito populations to dissect the mechanisms of resistance. Indeed, the cocktail of toxins produced by Bti, and especially the presence of Cyt toxin, appear to hinder the evolution of resistance to Bti both in the laboratory and in the field [2]. However, recent works conducted in our group revealed that Cyt1Aa toxin was strongly affected by the organic matter found in most mosquito breeding sites, inducing a decreased efficacy and a low-level persistence of Cyt toxins as compared to Cry toxins [10, 11]. This ultimately led to the modification of toxins proportions in field-persistent Bti (Cry toxins became the most abundant toxins while it is Cyt toxin in commercial Bti), as shown in leaf litters containing persistent Bti sampled in mosquito breeding sites several months after a treatment [11]. This field-collected Bti was used to select a laboratory strain for 22 generations, resistant to Bti toxins (LiTOX strain [12]). Two phenotypes were observed in the LiTOX strain: larvae with a normal development time (5 days as the parental Bora-Bora strain) named LiTOX_N, and larvae with a slow development (about 10 days) named LiTOX_S. As compared to the susceptible parental Bora-Bora strain, LiTOX_N exhibited resistance ratio (RR_50_) of 30.2-fold, 13.7-fold and 6.3-fold for Cry4Aa, Cry4Ba and Cry11Aa respectively. LiTOX_S exhibited the same resistance ratio as LiTOX_N for Cry4Ba and Cry11Aa but was not resistant to Cry4Aa [12]. In addition to an altered development, the resistant LiTOX strain exhibited lower female fecundity and lower egg survival, suggesting that Bti resistance is associated with high fitness costs [13]. In the present study, we used the LiTOX strain to undertake a whole transcriptome analysis by RNA-seq of the two developmental phenotypes LiTOX_S and LiTOX_N *versus* the susceptible parental strain aiming at dissecting the molecular pathways to resistance to Bti Cry toxins at the whole organism level, and at understanding the trade-offs underlying Cry-toxin resistance and life history traits. Transcription level and polymorphism variations associated with each resistance phenotype were identified in regards of known and new putative mechanisms involved in Bti toxins resistance and the way they might interfere with mosquito life history traits is discussed.

## Results

### Mapping and re-annotation results

A total of 197,175,220 short reads (75 bp) were sequenced, with an average of 33 million reads per sample. An average mapping rate of ~83% (164.66 million mapped reads) was reached. After filtering on base quality (150.86 million reads left) and on mapping quality, 129.32 million reads were retained for further analysis (Additional file 1: Table S1). A total of 12,942 transcripts showed a coverage >0.5 Reads Per Kilobase exon Model (RPKM) in at least one strain, including 2,717 transcripts showing a different structure as compared to genome annotation (21.8%) and 484 novel transcription events (NTEs) (3.7%) predicted based on spliced reads and distance from known transcripts (close or far, respectively). NTEs were not considered in further analyses. Because only 6,996 detected transcripts were functionally annotated in VectorBase (genome version AaegL2.1, vectorbase.org) we performed a Blast2GO analysis of all predicted peptides annotated as ‘conserved hypothetical protein’ or ‘hypothetical protein’ in Vectorbase against the protein database Swissprot (Blastp, E-value < 10^-3^, annotation cut-off = 55, GO weight = 5). This allowed re-annotation of 2,608 transcripts (Additional file 2: Table S2). We also re-annotated known mosquito Bti-toxin receptors based on bibliography [14, 15], ending up with about 77% of annotated transcripts in our dataset, including 80 putative Cry receptors (15 α-amylases, 14 ALPs, 12 cadherins and 39 APNs), and 40 trypsins/chymotrypsins potentially involved in Cry protoxin activation, and thereafter called ‘Bti candidates’. All the other transcripts were assigned to nine categories based on their annotation as follows: ‘Detoxification’, ‘Other enzymes’, ‘Cuticle’, ‘Immunity’, ‘Hormones and neurotransmitters signaling’, ‘Transcription factors’, ‘Intra or extracellular trafficking/chaperonins’, ‘Structure’ and ‘Unknown’.

### Differential expression

**Figure 1:**
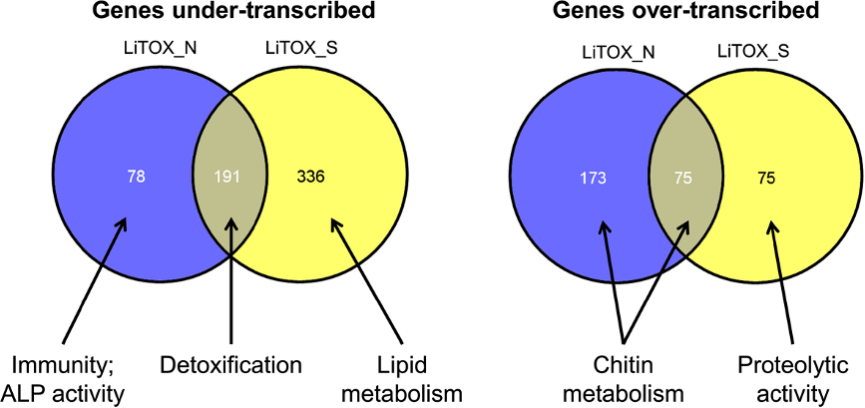
**Genes differentially expressed in LiTOX phenotypes.** For each Venn diagram section, the numbers of genes differentially expressed in each LiTOX phenotype as compared to the susceptible strain are indicated, together with the function(s) of these genes based on the list of GO terms significantly enriched (see Additional file 4: Table S4).

**Figure 2:**
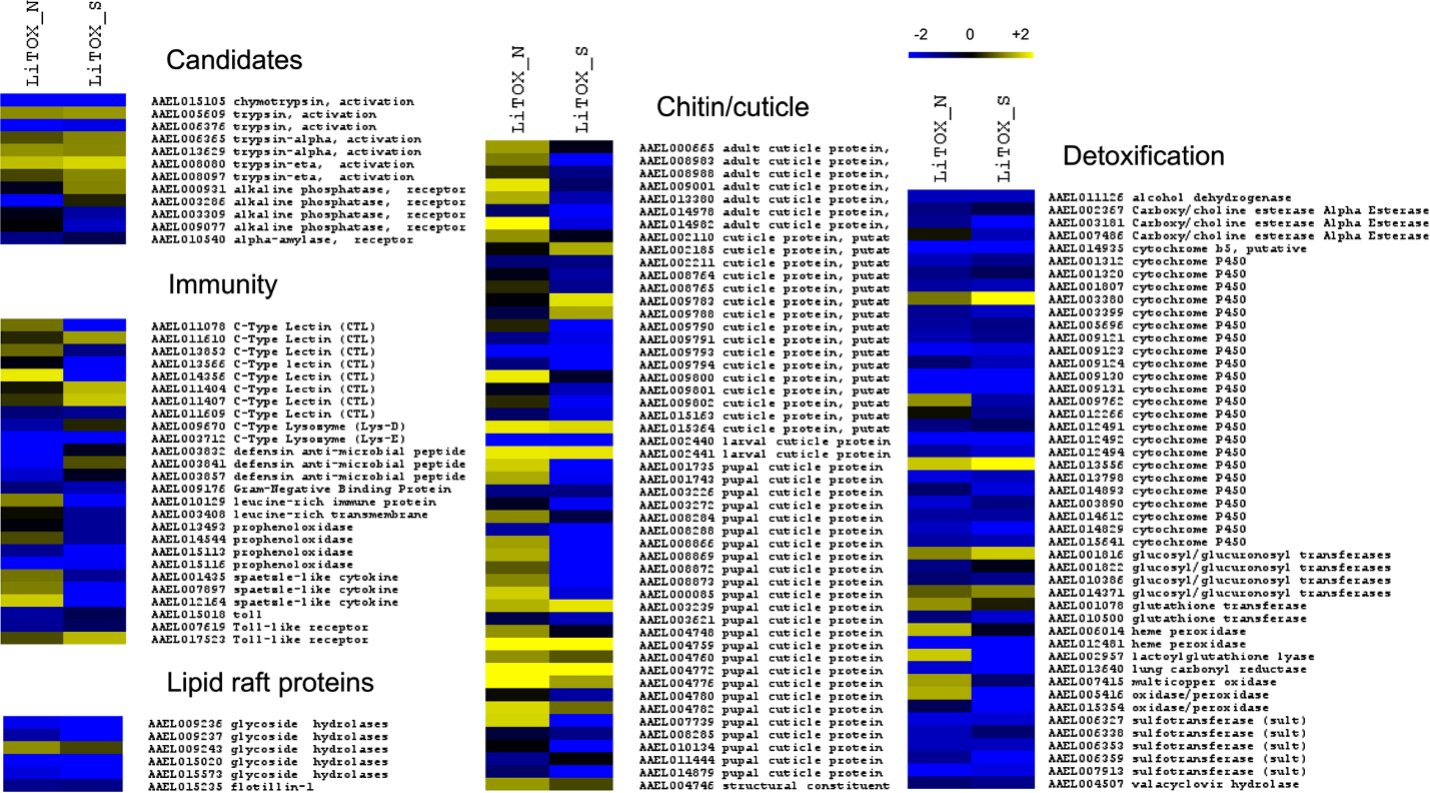
**Representation of genes significantly differentially transcribed in at least one LiTOX phenotype as compared to the susceptible strain for five functions: ‘Candidates’, ‘Lipid raft’, ‘Immunity’, ‘Cuticle’ and ‘Detoxification’.** The color scale corresponds to log_2_ transcription ratio as compared to the susceptible strain. Significant values in Additional file 3: Table S3.

**Figure 3:**
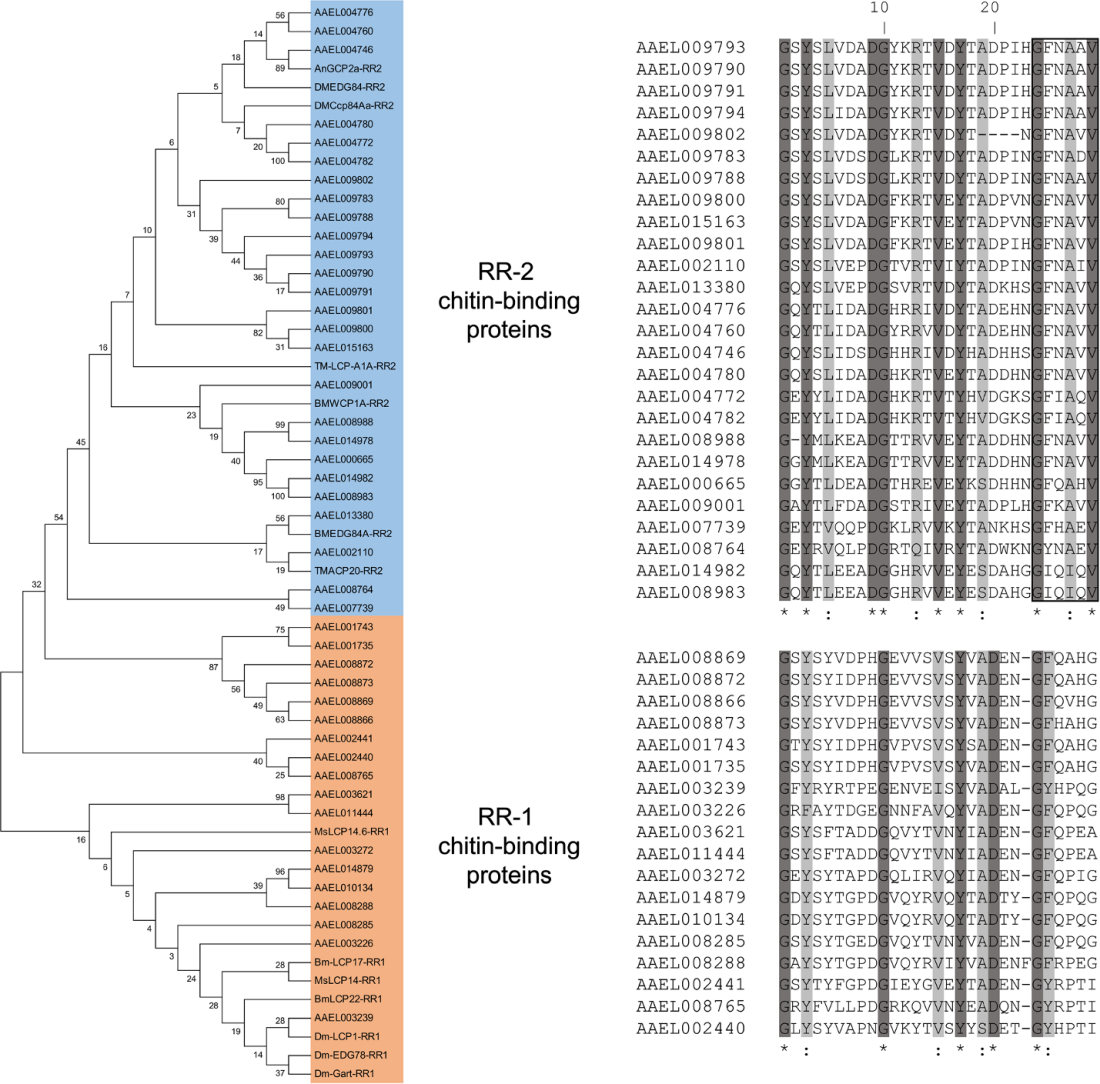
**Phylogenetic analysis and sequence alignment of R&R consensus domain from the 44 differentially transcribed genes encoding chitin-binding proteins.** Representatives from the two RR sub-groups from other insect species were added to support the tree (*Anopheles gambiae*: AnGCP2a-RR2, AAC05656; *Bombyx mori*: Bm-LCP17-RR1, FAA00504; BmLCP22-RR1, NP_001036828; BMEDG84A-RR2, BAA33195; BMWCP1A-RR2, BAB32475; *Drosophila melanogaster*: Dm-LCP1-RR1, NP_476619; Dm-EDG78-RR1, NP_524198; Dm-Gart-RR1, NP_476673; DMCcp84Aa-RR2, AAD19803; DMEDG84-RR2, P27780; *Manduca sexta*: MsLCP14-RR1, AAA29319; MsLCP14.6-RR1, Q94984; *Tenebrio molitor*: TM-LCP-A1A-RR2, P80681; TMACP20-RR2, P26967. Bootstrap values (2000 replicates) are shown on each branch of the tree. Symbols below the aligned amino acids indicate identity (*), or high conservation (:). Identical and highly conserved amino acids are highlighted in dark grey and light grey, respectively. The GFxAxV motif, diagnostic of the RR-2 sub-group, is highlighted by a box.

Among all detected genes, 844 (6.8%) were differentially transcribed (Table 1) in at least one LiTOX phenotype as compared to the parental susceptible strain, including 12 candidate genes (Additional file 3: Table S3). The two LiTOX phenotypes shared 266 differentially transcribed genes as compared to the susceptible (191 and 75 genes under and over-transcribed, respectively, Figure 1), and 84 genes were over-transcribed in LiTOX_N and under-transcribed in LiTOX_S. Among genes under-transcribed in both phenotypes, GO terms associated with detoxification process were over-represented, while those associated with cuticle/chitin metabolism were over-represented in genes over-transcribed (Figure 1, Additional file 4: Table S4). In genes specifically under-transcribed in LiTOX_N, GO terms associated with immunity and ALP activity were over-represented. In LiTOX_S, GO terms associated with lipid metabolism were over-represented among the under-transcribed genes and those associated with proteolytic activity were over-represented in over-transcribed genes. Among candidate genes, some specific proteases such as the chymotrypsin AAEL015105 or the trypsin AAEL006376, potentially involved in Cry protoxin activation, were under-transcribed in both LiTOX phenotypes (Figure 2). Among genes differentially transcribed between the two LiTOX phenotypes, one serine protease (AAEL010139) was below the detection threshold in LiTOX_N (0.2 RPKM, as compared to 7.4 and 8.2 RPKM in Bora-Bora and LiTOX_S strains respectively), and five putative Cry receptors were differentially transcribed: one α-amylase (AAEL010540) and four ALPs (AAEL003286, AAEL003309, AAEL009077 and AAEL000931). Genes involved in immunity showed contrasted expression patterns in the two LiTOX phenotypes: several genes involved in the melanization process (prophenoloxidases) were under-transcribed in both LiTOX phenotypes, three defensins (antimicrobial peptides) were under-transcribed only in LiTOX_N, and several genes involved in the Toll pathway (e.g., spaetzle-like cytokine) were over-transcribed in LiTOX_N and under-transcribed in LiTOX_S. Finally, genes involved in cuticle/chitin metabolism were mostly over-transcribed in LiTOX_N and under-transcribed in LiTOX_S. Conserved domain search (CDS) revealed that all but four were chitin-binding proteins containing the typical Rebers & Riddiford (RR) consensus motif [16]. Based on phylogenetic analysis and sequences alignment, 26 (59%) contained the diagnostic motif GFxAxV (degenerated in three of them) of RR-2 cuticle proteins while 18 (41%) were RR-1 cuticle proteins [17, 18] (Figure 3).

**Table 1:** **Differential transcription analysis overview: Number (and proportion) of transcripts significantly over or under-transcribed in each LiTOX phenotype and altogether (LiTOX-N or LiTOX-S) as compared to the control parental strain**

	LiTOX-N		LiTOX-S		Any strains	
	Transcripts	%	Transcripts	%	Transcripts	%
AC* test P value and FC > =2	564	4.4%	745	5.8%	923	7.1%
Over transcribed	274	2.1%	166	1.3%	356	2.8%
Known transcripts	248	1.9%	150	1.2%	323	2.5%
Novel transcription event	26	0.2%	16	0.1%	33	0.3%
Under transcribed	290	2.2%	579	4.5%	661	5.1%
Known transcripts	269	2.1%	528	4.1%	605	4.7%
Novel transcription event	21	0.2%	51	0.4%	56	0.4%

*Audic-Clavery test.

### SNPs detection and analysis

**Figure 4:**
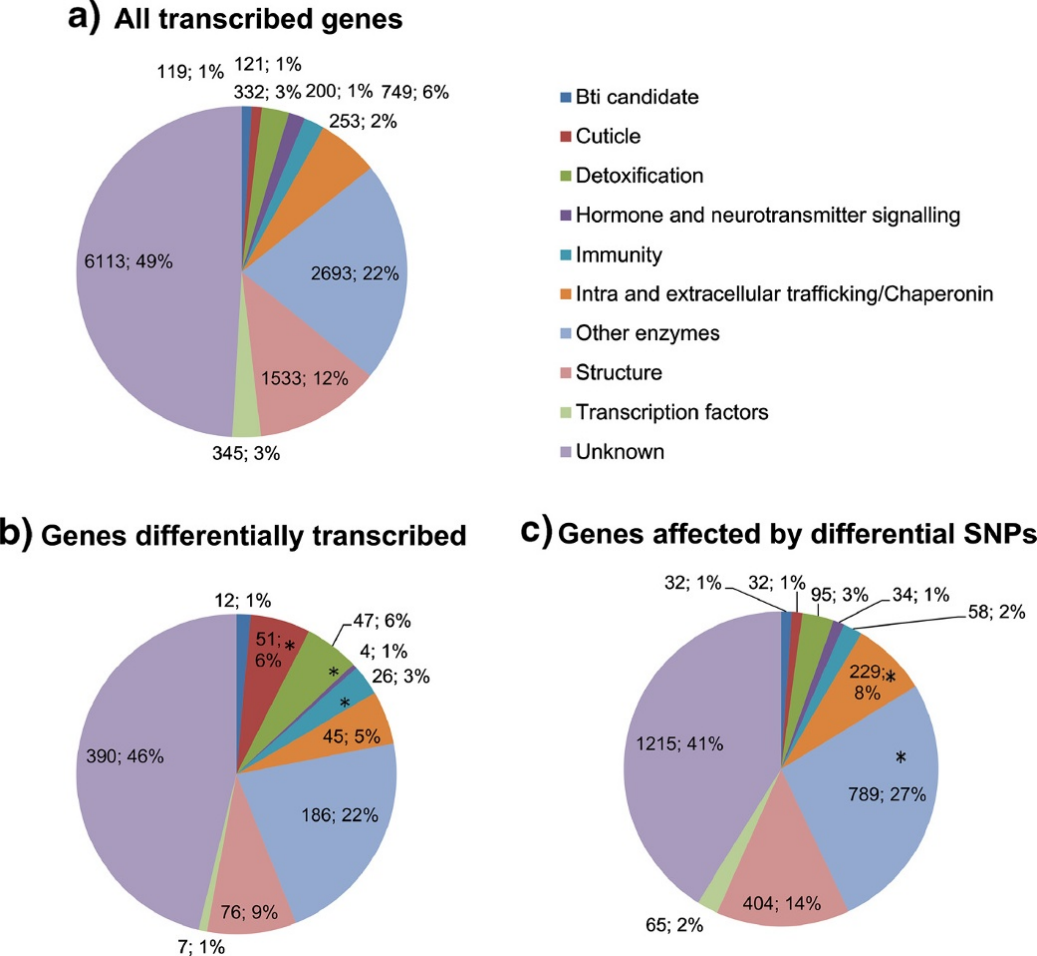
**Biological functions of a) all detected genes (12,458), b) of genes differentially transcribed (844 genes) and c) of genes affected by differential SNPs between the LiTOX and the susceptible strains (2,953 genes).** Genes were categorized into 10 categories: Categories were defined as follow: ‘Candidate’, ‘Detoxification’, ‘Other enzymes’, ‘Cuticle’, ‘Immunity’, ‘Hormones and neurotransmitters signaling’, ‘Transcription factors’, ‘Intra or extracellular trafficking/chaperonins’, ‘Structure’ and ‘Unknown’. Enrichment of these categories compared to all detected transcripts was then computed using a one-side Fisher’s exact test followed by Benjamini and Hochberg multiple test correction. Categories showing a corrected P value <0.05 are indicated with a star.

**Figure 5:**
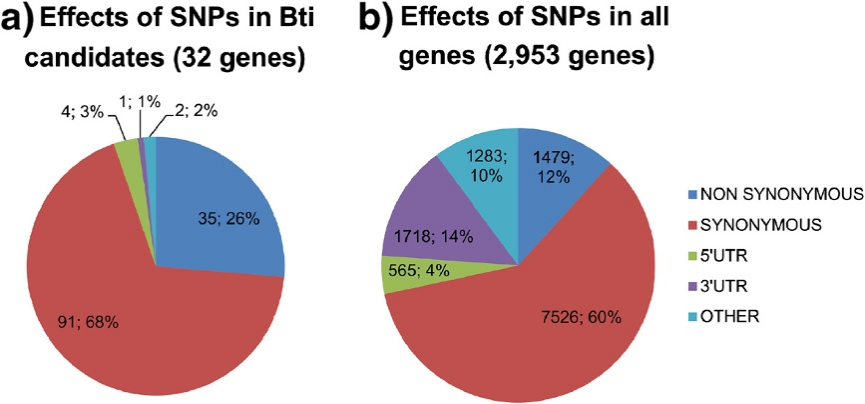
**Distribution of the effects of differential SNPs a) in the 32 candidate genes containing differential SNPs and b) in all 2,953 genes containing differential SNPs.** SNP effects were categorized into ‘non-synonymous’, ‘synonymous’, ‘3’UTR’, 5’UTR’, or ‘other’.

A total of 166,943 SNPs were called, of which 68,541 had a total coverage ≥30 and affected 6,511 genes distributed over ~67% (1106/1636) of the *Ae. aegypti* supercontigs that have annotated genes, including 66 candidate genes (50 receptors and 16 trypsins/chymotrypsins). A total of 2,953 genes were affected by 12,571 differential SNPs (more than 40% allelic frequency difference between the susceptible and resistant strains). Little overlap was found between genes differentially transcribed in selected strains and those affected by differential SNPs: only 76 differentially expressed genes were affected by non-synonymous differential SNP (Additional file 3: Table S3). This was expected as RNAseq data are restricted to transcripts and did not cover regulatory regions often located outside transcript boundaries. While in genes differentially transcribed, three categories of genes were over-represented (‘chitin/cuticle’, ‘detoxification’ and ‘immunity’), two other categories of genes (‘intra/extra cellular trafficking’ and ‘enzymes’) were over-represented in genes affected by differential SNPs between the resistant and susceptible strains (Figure 4). Thirty two candidates (25 putative toxin receptors and 7 trypsins) were affected by a total of 508 SNPs from which 133 (26.3%) were differential SNPs; although this is not significantly different from the proportion of differential SNPs in all other genes affected, non-synonymous mutations were significantly more abundant in candidates than in any other affected genes (Fisher exact test, *p* = 10^-4^; Figure 5). Among the putative Cry receptors, one cadherin, 12 aminopeptidases and 8 α-amylases were affected by differential SNPs (Additional file 5: Table S5). This included 27 non-synonymous SNPs affecting the protein sequence of 1 cadherin, 8 APNs and 4 α-amylases (Table 2). There was little difference in sequence between the two resistant phenotypes, with only 330 differential SNPs (including 14 non-synonymous changes) affecting 130 genes, none of them being candidate genes (Additional file 6: Table S6).

**Table 2:** **Non-synonymous changes in putative Cry toxin receptors, with their frequency and coverage in the susceptible and resistant phenotypes**

				Bora		LiTOX_N		LiTOX_S					
Receptor	Gene ID	Reference	Variant	%	Coverage	%	Coverage	%	Coverage	Position cDNA	Amino acid position	Amino acid change	Toxin ^1^
APN1	AAEL009108	C	A	4.1	145	55.1	98	51.9	106	2539	847	A- > S	
APN2	AAEL003227	C	T	26.6	30	63.6	11	88.9	9	2240	747	T- > I	
APN2	AAEL005808	C	A	30.4	46	73.3	45	91.4	70	2289	763	M- > I	Cry4Ba [40]
	AAEL005808	C	T	31.4	51	85.7	91	95.5	110	2725	909	V- > I	Cry4Ba [40]
	AAEL005808	C	T	15.5	103	77.7	130	75.7	107	1762	588	A- > T	Cry4Ba [40]
	AAEL005808	T	G	14.5	117	84.7	111	89.4	132	1145	382	E- > A	Cry4Ba [40]
APN2	AAEL005821	A	G	5.5	255	50.0	82	63.3	150	1660	554	T- > A	
	AAEL005821	T	C	5.0	260	56.1	98	65.0	183	1646	549	M- > T	
	AAEL005821	G	A	10.1	179	71.6	88	78.5	158	1459	487	D- > N	
APN4	AAEL007204	C	T	20.2	263	67.4	307	70.7	314	167	56	T- > M	
	AAEL007204	G	C	40.0	190	93.2	294	95.8	239	216	72	E- > D	
	AAEL007204	C	T	33.8	142	93.9	197	92.7	220	283	95	H- > Y	
APN4	AAEL012774	C	T	43.4	459	88.3	677	86.9	540	311	99	G- > R	Cry11Aa [46]
APN5	AAEL002696	A	G	23.1	65	56.3	48	64.6	65	472	158	K- > E	
	AAEL002696	G	A	57.7	71	100.0	66	100.0	59	2380	794	V- > M	
	AAEL002696	C	A	17.8	90	66.7	42	60.4	48	2432	811	A- > E	
	AAEL002696	G	A	12.3	57	70.5	44	70.0	50	746	249	R- > K	
APN5	AAEL003666	A	G	24.6	126	63.9	72	70.8	106	1814	605	V- > A	
	AAEL003666	C	A	16.9	83	64.9	74	73.0	89	595	199	A- > S	
APN7	AAEL007201	A	G	25.2	163	76.1	46	96.4	28	2219	740	N- > S	
Cadherin	AAEL018140	T	A	12.1	33	43.9	57	58.7	63	790	160	H- > L	Cry11Aa [15]
α-amylase	AAEL000642	A	T	24.5	49	67.9	56	47.0	66	1424	468	T- > S	
α-amylase	AAEL007673	C	T	18.0	589	51.3	493	75.0	559	47	16	S- > N	
α-amylase	AAEL010537	AA	TT	12.2	395	49.7	447	55.4	482	1706	564	I- > F	
	AAEL010537	A	G	15.6	825	53.0	1078	58.7	1368	1324	437	K- > R	
	AAEL010537	C	T	16.0	268	60.5	248	64.3	412	18	2	R- > W	
	AAEL010537	C	T	21.7	1154	76.1	804	83.0	833	1552	513	A- > V	
α-amylase	AAEL014710	G	A	9.9	81	60.0	35	56.5	23	992	331	A- > V	

^1^Toxin shown to bind this receptor in the literature.

## Discussion

Two main mechanisms of resistance to Cry toxins have been described in insects so far: altered protoxin activation by gut proteases, and modification in transcription level and/or protein sequence of Cry receptors resulting in lower or failure in toxin binding [19–21]. The study of resistance to Cry toxins in insects has mostly been based on the study of a few candidate proteins first identified in lepidopterans, either toxin receptors or proteases, using proteomic approaches (2D-DIGE, ligand blotting, etc.). As compared to other *Bacillus thuringiensis* subspecies that typically produce a single Cry toxin, Bti produces a mixture of four different toxins, and resistance to Bti is likely to involve multiple distinct mechanisms, which may affect the physiology and metabolism of resistant insects. Altered metabolism in Bti resistant mosquitoes was revealed by fitness costs expressed at any mosquito life-stage (larval development time, fecundity, egg survival) [13]. These costs can result from pleiotropic effects of resistant alleles on life-history traits (direct cost) or more indirectly from resource reallocation between different metabolic pathways.

### Global changes in gene expression and metabolic trade-offs

The two LiTOX phenotypes share common global transcriptional responses: an over-transcription of genes involved in proteolytic activity, cell cytokinesis and wound healing, and an under-transcription of enzymes classically involved in the detoxification of chemicals such as chemical insecticides, plants allelochemicals and pollutants (cytochrome P450 monooxygenases, esterases, glutathion S-transferases, UDP-glycosyltransferases, etc.). Conversely, Bti toxins are large proteins and their degradation requires a proteolytic processing rather than the activity of detoxification enzymes. Therefore, in the absence of chemical selection pressure, the under-transcription of these enzymes might represent a metabolic trade-off in the LiTOX strain to compensate the increased expression of enzymes involved in protein degradation [22]. The over-transcription of several proteases (endopeptidases, serine proteases) is in accordance with an observed increase in proteolytic activities of midgut enzymes of LiTOX larvae [23]. This increased proteolytic activity in Bti resistant larvae could reflect a higher ability to degrade toxins. Despite this global trend, particular proteases such as the metalloproteinases AAEL011547, AAEL011550 and AAEL014514, and the chymotrypsin AAEL015105 or the trypsin AAEL006376, which are putatively involved in Cry protoxin activation, were under-transcribed in both LiTOX phenotypes. The lack of metalloproteinase activity in the LiTOX strain was evidenced by the analysis of enzymatic activities in the midgut of larvae using various specific enzyme inhibitors [22]. Down-regulation of specific proteases might result in a lower and/or improper toxin activation which could also confer resistance. In lepidopterans, analysis of proteinase activities from gut extracts have shown the lack of trypsin-like and of chymotrypsin-like activity in Bt-resistant strains [20]. Furthermore, the role of proteases in resistance to Bt toxin Cry1Ca1 was demonstrated in the caterpillar *Spodoptera frugiperda* where a serine-protease gene under-transcribed in the midgut of intoxicated larvae was further shown by RNAi-mediated knockdown to be involved in both reduced protoxin activation in the midgut and reduced susceptibility of caterpillars to toxins in bioassays [24].

### Lipid rafts and Bti-toxins trafficking through the gut membrane

Lipid rafts are membrane domains enriched in glycosylphosphatidylinositol (GPI)-anchored proteins that have been implicated in transmembrane protein trafficking and were shown to play a role as portals of entry for many pathogens including viruses, bacteria and their toxins [25, 26]. Several studies suggested that lipid rafts might play a central role in Bt-toxins toxicity. For example, the presence of lipid rafts was shown to be essential for the toxicity of Cry1C on Sf9 Lepidoptera cells [27]. However, the specific proteins associated with this mechanism have not been identified yet. A flotillin, a protein found in lipid rafts and described as a toxin transporter [28], was under-transcribed in both LiTOX phenotypes. Flotillin was shown to co-localize with Cry4Ba toxin in *Aedes aegypti* larval gut brush border membranes [25], and further investigation of its Cry-binding ability will contribute to characterize its role in Bti toxin resistance. Four glycoside-hydrolases were under-transcribed in both LiTOX phenotypes. Although never described as Cry toxin receptors, glycoside-hydrolases were shown to be associated with lipid rafts in the gut membrane of *Aedes* larvae [25]. Lipid rafts appear to play a central role in pore-forming toxins insertion in the gut membrane by functioning as platforms to recruit GPI-anchored proteins including APNs, ALPs, and glycoside-hydrolases. The role of some GPI-anchored proteins (flotillin and glycoside-hydrolases) largely present in lipid rafts of *Aedes* larvae guts has been so far underexplored in studies aiming at understanding Cry toxins toxicity in insects, and the present study pinpoints them as new potential candidates in *Aedes*.

### Differences between the two LiTOX phenotypes

Several gene families were differentially transcribed between the two developmental phenotypes: genes involved in cuticle/chitin metabolism, prophenoloxidases, and genes coding for enzymes involved in lipid metabolism.

In our dataset, the category of cuticle/chitin proteins contained the highest number of genes differentially transcribed (Figures 2 and 4b, Additional file 3: Table S3). Cuticle is an exoskeleton constituted of chitin that provides physical support and protects the insects from physical injuries, desiccation and infections. Cuticle is typically divided into hard cuticle, generally sclerotized and mechanically stiff, and soft cuticle, a more flexible region that allows locomotion and appendix movements [29]. Chitin is always associated with chitin-binding proteins; although their exact function is to be investigated, it has been hypothesized that they could play important roles in cuticle metabolism and structure preservation [30]. Nearly all the genes identified as differentially transcribed in this category contained the R&R chitin-binding motif and were associated with the soft cuticle (RR-1 cuticle proteins) or with the hard cuticle (RR-2) [17]. The global under-transcription of these genes in the LiTOX_S could partly explain the slow development of this cohort, considering that a low chitin metabolism is believed to slow-down the molting process and increase the development duration. Nevertheless, it is intriguing why only genes encoding R&R-containing proteins were found differentially transcribed while genes encoding other chitin-binding proteins with peritrophin-A chitin-binding domains (ChtBD2 domain) and chitin metabolism enzymes (such as chitinases) were not differentially expressed. Also, it is challenging to know if this pattern is a side-effect consequence of the resistance phenotype or if it is part of the mechanisms of resistance developed by the insects against Cry toxins. A better understanding of the role of such proteins in the cuticle metabolism is mandatory to help deciphering their potential involvement in resistance phenotypes in insects.

Prophenoloxidases are involved in the melanization process, and as such they have a dual role: they contribute to cuticle formation (and larval development), and they participate in the immune response against pathogens by their encapsulation [31]. In *Ae. aegypti*, both defensins and phenoloxidases were shown to participate to immune response against bacterial challenge [32]. In our dataset, prophenoloxidases were under-transcribed in both LiTOX phenotypes, while defensins were under-transcribed only in LiTOX_N (Figure 2, Additional file 3: Table S3). Furthermore, a correlation was observed between age at pupation (development speed) and mosquito's ability to melanize Sephadex beads (immuno-capacity), suggesting that an increased immunity is costly and results in a slower development [33]. The two developmental phenotypes observed in the resistant mosquito strain LiTOX might be the result of a trade-off between immunity and development.

When focusing on candidate genes, only few differences in transcription level and no significant difference in polymorphism were observed between the two LiTOX phenotypes (e.g. between LiTOX_N and LiTOX_S). The serine protease AAEL010139 was not expressed in LiTOX_N, the Cry4Aa-resistant cohort. Among the putative Cry-toxin receptors, four alkaline phosphatases (ALPs) were differentially transcribed between the two LiTOX phenotypes: ALP AAEL003286 was specifically under-transcribed in LiTOX_N, which may be linked to the high Cry4Aa resistance of this phenotype. Three other ALP were specifically under- (AAEL003309 and AAEL009077) or over-transcribed (AAEL000931) in LiTOX_S. Cry4Aa mode of action and mechanisms of resistance are the least investigated and understood among the Bti toxins. Functionally validating the involvement of these few candidate genes could help lifting the veil on the specific mechanisms associated with Cry4Aa resistance and provide a better understanding of the patterns of cross-resistance between Bti Cry toxins.

### Polymorphisms rather than expression changes are found in putative Bti-toxins receptors

Among the 80 putative Cry receptors detected in larvae, only 5 were differentially transcribed in the LiTOX strain as compared to the susceptible strain. Among these, the α-amylase AAEL010540 was under-transcribed in both LiTOX phenotypes sharing resistance to Cry4Ba and Cry11Aa. In *An. albimanus*, an α-amylase (aamy1) has been described as a Bti toxin receptor, binding both Cry4Ba and Cry11Aa [9]. Although this requires functional validation, this suggests that the *Ae. aegypti* α-amylase AAEL010540 (48% identity with aamy1) could bind Cry toxins. No cadherin or APN that were previously associated with Cry resistance in insects [34–37] or showed to bind Bti Cry toxins in mosquitoes [38–40] were differentially transcribed in the LiTOX strain, suggesting that if they are involved in Bti resistance in LiTOX, this is not related to an altered expression but rather to changes in protein sequence affecting their affinity for Cry toxins. Indeed, one cadherin, eight APNs and four α-amylases displayed differential SNPs leading to non-synonymous changes between the resistant and susceptible strains.

#### Cadherin

CAD-like proteins have been the most intensively studied putative Cry toxin receptor molecules in lepidopteran and coleopteran larvae [41–43]. It has been proposed that they act as the first receptors of Cry toxins, binding toxin monomers and facilitating further processing required for the pre-pore oligomer formation [44]. Although twelve CAD-like proteins were detected in our dataset, none was differentially transcribed, and only one (AAEL018140) contained several differential SNPs (Table 2). This cadherin was previously shown by proteomic approaches to act as a Cry11Aa toxin receptor [15]. It contains a N-terminal signal peptide, 11 cadherin repeats (CR1 to CR11), a membrane-proximal region (MPR) and a trans-membrane domain. The only differential non-synonymous SNP distinguishing Bti-resistant and susceptible strains (H160L) is located in the N-terminal region preceding the first cadherin repeat (CR1), while overlay assays and immune-blotting localized Cry11Aa and Cry4Aa toxin-binding regions in CR8-CR11 [15]. More generally, Cry-toxin binding sites were usually described in the membrane-proximal region of insect cadherins, not in the N-terminal extracellular region [8]. However, amino-acid substitution at any location can dramatically change the secondary structure of a protein; for instance, the three cadherin alleles conferring resistance to Bt in the pink bollworm *Ectinophora gossypiella* are not located within the Cry1Aa binding site but upstream [45]. In this case, the deletion of several amino-acids upstream the toxin binding site was shown to alter the full-length cadherin and impede toxin binding. In the H160L mutation observed at relatively high frequency in the LiTOX strain (49-58%), a polar and positively charged amino acid (Histidine) is replaced by a non-charged hydrophobic amino acid (Leucine), which can result in a change in the spatial arrangement of the cadherin in the cell membrane. Further functional studies and *in-silico* protein modelling could allow investigating whether this change affects cadherin conformation and Cry toxin binding affinity. Altogether, our results suggest that cadherin might not have a central role in resistance to Bti in mosquitoes.

#### APNs and α-amylases

APNs and α-amylases are digestive enzymes that play a key role in larval nutrition and development, being involved in protein and glucose metabolism, respectively. APNs have been extensively studied as putative Cry toxin receptors in many insects and were classified into 8 classes based on their sequence identity [8, 14]. APNs from several classes were reported to bind with more or less affinity to the different Cry toxins in various insects, including AeAPN1 in *Aedes* and APN2 in *Anopheles* [14], but their precise role in Bti toxicity remains to be elucidated. Out of the 31 APNs detected in *Ae. aegypti* larvae, 12 contained differential SNPs, and non-synonymous differential changes affected the sequence of 8 of them (Table 2). In the APN2 AAEL005808 recently shown to be a functional receptor of Cry4Ba toxin [40], 4 non-synonymous changes were nearly fixed in the LiTOX strain (both phenotypes), suggesting ongoing selective sweep on this gene. One non-synonymous change affected the sequence of the APN1 AAEL012778 previously shown to bind Cry11Aa [46]. Although not usually cited as a Cry-toxin receptor, an α-amylase was recently shown to bind Cry4Ba and Cry11Aa in *Anopheles* [9]. Our dataset pinpoints five α-amylases potentially involved in *Aedes* resistance to Bti toxins: one α-amylase was under-transcribed only in LiTOX_N (resistant to all Cry toxins), and four other α-amylases presented sequence changes in both cohorts of the resistant LiTOX strain as compared to the susceptible strain. Because of their key role in insect nutrition, altered expression of these digestive enzymes as a resistance mechanism is likely to be costly in terms of larval development, and this might explain why the observed changes in these enzymes are mostly amino-acid changes rather than changes in expression levels. Indeed, change in the protein sequence might dramatically alter toxin binding site while having small effects on digestive efficiency. Further studies on the differential abilities of these α-amylases to bind Bti Cry toxins and to act as functional toxin receptors are required to determine their role in Bti toxicity. Also, molecular modelling of APN is necessary to identify the oligosaccharide that are involved in the binding of Cry toxins to verify if the SNPs described in this study can alter the Cry-APN interaction and partly explain the resistance phenotype observed [47].

## Conclusions

The present study is the first to analyze the whole transcriptome of Bti-resistant larvae by deep sequencing, allowing studying change in gene expression level and sequence polymorphisms using the same dataset. It reveals dramatic modifications in the transcriptional profiles selected in resistant larvae, with a global transcriptional increase of genes coding for proteases and chitin-binding proteins, and a decrease in transcription of genes involved in detoxification and immunity. Whether these modifications are directly involved in Bti-toxin processing or more indirectly through complex metabolic compensations selected to limit the cost of resistance remains to be functionally investigated. Regarding candidate Bti-toxin receptors, our dataset relativizes the role of cadherin in resistance to Bti in mosquitoes, and highlights the importance of studying changes in the sequence rather than in transcription levels of APN and ALP, but also of other proteins involved in protein trafficking through the cell membrane such as flotillins and glucoside hydrolases.

## Methods

### Strains and Sample preparation

The *Ae. aegypti* laboratory strain Bora-Bora susceptible to all insecticides was selected using field-collected leaf-litter containing *Bti* toxins (LiTOX strain) as described in [12]. At each generation, an average of 6,000 larvae was exposed to toxic leaf litter in order to obtain a mortality rate of 70%, so that about 1,800 adults constituted the next generation, limiting bottleneck effects in the LiTOX strain. Mosquito strains were reared in standard insectarium conditions (27°C, 14 h/10 h light/dark period, 80% relative humidity). The average generation turnover was 45 days and selection was carried out over 18 generations. Larvae were bred in the insectarium till they reached early 4^th^ instar. LiTOX_S larvae were allowed to develop for 5 days more than other larvae. For each of the three phenotype (Bora-Bora, LiTOX_N and LiTOX_S) total mRNA was extracted from three pools of 60 4th-stage larvae (reared in different batches and originating from different egg-laying female pools) at the same time of the day, using the RNAqueous-4PCR kit (Applied Biosystems/Ambion, Austin, TX, USA), according to the manufacturer’s instructions. RNA quantity and quality was checked using bioanalyzer, and RNA from the 3 biological replicates was pooled in equal proportion to obtain one representative total RNA sample per phenotype (each made from 180 individuals). For each of the three phenotypes, two distinct cDNA libraries were prepared from the same pool of total mRNA and sent at the National Genotyping Center (Genoscope, France) and sequenced individually on the Illumina Genome Analyser II system (GAII) to assess technical variations.

### Short read mapping and assembly on the reference genome

The Tophat algorithm (release 2.0.9, http://ccb.jhu.edu/software/tophat) was applied with defaults parameters to align all the reads onto the *Aedes aegypti* reference genome AaegL2.1 (http://ccb.jhu.edu/software/tophat) by taking into account, both already known (--no-novel-juncs) and novel *ab initio* splice exon-exon junctions [48].

### Estimation of transcripts’ relative abundance and differential expression (DE) analysis

Bam files were then loaded into Genespring NGS Version 12 (Agilent) software. Reads were filtered on base quality (mean base quality >30 and <10 N per reads) and on mapping quality (alignment score > =97 and Mapping quality > = 250 and remove non-primary multiply mapped reads). *Ae. aegypti* genome was then re-annotated based on reads distribution and RPKM calculated for each known or new putative transcript using default parameters (min exon length percentile 10, min intro length percentile 10, max intron length percentile 90, min exon RPKM percentile 50, min gene RPKM percentile 50, min gene length percentile 10, min exon RPKM with respect to host gene percentage 75, minimum number of reads in exon 10). For each strain, RPKM correlation between technical replicates was calculated. As r^2^ were >0.9 for all strains (acceptable technical variation, see Additional file 7: Figure S1), technical replicates were pooled for further analyses, in order to reach a high coverage per transcript and per SNP position. Transcripts with >0.5 RPKM in at least one strain were retained for differential transcription analysis. Differential transcription level was tested for each transcript between each resistant phenotype (LiTOX_N and LiTOX_S) and the parental strain Bora-Bora using an Audic Clavery test designed to compare two cDNA libraries [49]. This Bayesian method is based on the assumption that under the null hypothesis, read counts of the same gene in two libraries come from the same but unknown Poisson distribution. The posterior probability is obtained by Bayesian averaging (infinite mixture) of all possible Poisson distributions with mixing proportions equal to the posteriors under the flat prior. This probability is further adjusted for multiple testing (Benjamini and Hochberg correction). Despite this popular method has been validated by the statistician community [50], we recommend sequencing biological replicates rather than pooling them prior sequencing in future RNAseq analyses, as sequencing costs are now acceptable and the later approach allows a more robust identification of genes affected by variations across conditions. Transcripts showing an adjusted P- value <10^-15^ and a fold change ≥2 fold in either direction were considered differentially transcribed.

### GO term analysis

The annotation terms from the GO ontology were retrieved from the VectorBase-UniprotKB links. For each resistant phenotype and each differential expression state (i.e. up or down regulated as compared to the Bora-Bora strain), GO terms significantly over-represented were determined using a corrected P-value threshold of 0.05.

### SNP detection and analysis

Detection of polymorphisms was performed based on the 129.32 million reads passing quality filters with the following parameters: confidence score threshold =100, coverage >20 reads, base quality cut off =5, ignore locations within or next to homopolymer stretches >10 nucleotides. Among all detected polymorphism variations, only SNP substitutions were considered for differential polymorphism analyses. SNP allele frequencies were then computed between each LiTOX phenotype and the susceptible strain. In a previous study on the LiTOX strain, we have validated the reliability of mRNA sequencing pooled data for inferring population allelic frequencies on 269 SNP: the correlation between allelic frequencies obtained from pooled mRNA sequencing (180 pooled larvae) and individual genotypes (N = 28 individuals) obtained using a DNA Illumina GoldenGate array was highly significant (P <0.001, r =0.85) [51]. Allele frequencies were considered as differential between a LiTOX phenotype and the susceptible strain (hereafter named as differential SNPs) if the following conditions were fulfilled: total read coverage at SNP position ≥30 and allelic frequency difference between both strains ≥40% in either direction. Genic effects of SNPs were computed by comparing SNP locations with reference genome annotation, and were defined as 5’UTR, synonymous, non-synonymous, 3’UTR and other (intronic or intergenic- *i.e.* close but not within gene boundaries).

### Phylogenetic analysis and sequence alignment of R&R consensus domain from the differentially transcribed genes encoding chitin-binding proteins

Phylogenetic analysis (neighbor joining) was performed using MEGA 6.06 software on the chitin-binding domains identified using the CDS search software (https://www.ncbi.nlm.nih.gov/Structure/cdd/wrpsb.cgi). Representatives from the two RR sub-groups from other insect species were added to support the tree (*Anopheles gambiae*: AnGCP2a-RR2, AAC05656; *Bombyx mori*: Bm-LCP17-RR1, FAA00504; BmLCP22-RR1, NP_001036828; BMEDG84A-RR2, BAA33195; BMWCP1A-RR2, BAB32475; *Drosophila melanogaster*: Dm-LCP1-RR1, NP_476619; Dm-EDG78-RR1, NP_524198; Dm-Gart-RR1, NP_476673; DMCcp84Aa-RR2, AAD19803; DMEDG84-RR2, P27780; *Manduca sexta*: MsLCP14-RR1, AAA29319; MsLCP14.6-RR1, Q94984; *Tenebrio molitor*: TM-LCP-A1A-RR2, P80681; TMACP20-RR2, P26967). Alignments were performed using the ClustalW software from PBIL Expasy tool (http://npsa-pbil.ibcp.fr/cgi-bin/npsa_automat.pl?page=/NPSA/npsa_clustalw.html).

### Ethics

Mosquito rearing was performed according to protocol CEEA-LR-13002 agreed by the French National Committee of ethics for animal experimentation.

## Data access

RNAseq data are available at EBI/SRA/ArrayExpress (accession number E-MTAB-1635).

## Electronic supplementary material

Additional file 1: Table S1: Sequencing and mapping statistics. (XLSX 15 KB)

Additional file 2: Table S2: Blast2GO analysis of all predicted peptides annotated as ‘conserved hypothetical protein’ or ‘hypothetical protein’ in Vectorbase against the protein database Swissprot (Blastp, E-value < 10-3, annotation cut-off = 55, GO weight = 5). (XLSX 134 KB)

Additional file 3: Table S3: List of genes differently expressed between resistant and susceptible Bora-Bora strains classified according to their category. (XLSX 2 MB)

Additional file 4: Table S4: GO terms enrichment analysis. Analysis was performed on transcripts significantly differentially expressed in each LiTOX phenotype as compared to the susceptible strain. GO terms associated with each transcript were extracted from Vectorbase. GO terms showing adjusted P values <0.05 were considered significantly enriched. (XLSX 17 KB)

Additional file 5: Table S5: List of supercontigs and genes affected by differential SNPs and their effects. The total number of SNPs affecting these genes is also shown. (XLSX 315 KB)

Additional file 6: Table S6: List of genes with differential SNPs between LiTOX_N and LiTOX_S; non-synonymous changes are indicated. (XLSX 19 KB)

Additional file 7: Figure S1: RPKM correlations between cDNA library replicates. Each dot represents one transcript. Only transcripts showing more than 0.5 RPKM are shown. (XLSX 2 MB)
